# *N*-(2-Allyl-4-chloro-2*H*-indazol-5-yl)-4-meth­oxy­benzene­sulfonamide hemi­hydrate

**DOI:** 10.1107/S1600536813020606

**Published:** 2013-07-31

**Authors:** Hakima Chicha, Assoman Kouakou, El Mostapha Rakib, Mohamed Saadi, Lahcen El Ammari

**Affiliations:** a Laboratoire de Chimie Organique et Analytique, Université Sultan Moulay Slimane, Faculté des Sciences et Techniques, Béni-Mellal, BP 523, Morocco; b Laboratoire de Chimie du Solide Appliquée, Faculté des Sciences, Université Mohammed V-Agdal, Avenue Ibn Battouta, BP 1014, Rabat, Morocco

## Abstract

The fused five- and six-membered rings in the title compound, C_17_H_16_ClN_3_O_3_S·0.5H_2_O, are practically coplanar, with the maximum deviation from the mean plane being 0.057 (3) Å for the C atom bound to the exocyclic N atom. The indazole system makes a dihedral angle of 66.18 (12)° with the plane through the benzene ring, and it is nearly perpendicular to the allyl group, as indicated by the N—N—C—C torsion angle of 79.2 (3)°. In the crystal, the water mol­ecule, lying on a twofold axis, forms O—H⋯N and accepts N—H⋯O hydrogen bonds. Additional C—H⋯O hydrogen bonds contribute to the formation of a chain along the *b-*axis direction.

## Related literature

For the pharmacological activity of sulfonamides, see: Brzozowski *et al.* (2010 ▶); Drew (2000 ▶); Garaj *et al.* (2005 ▶); Lopez *et al.* (2010 ▶). For similar compounds, see: Abbassi *et al.* (2012 ▶, 2013 ▶). 
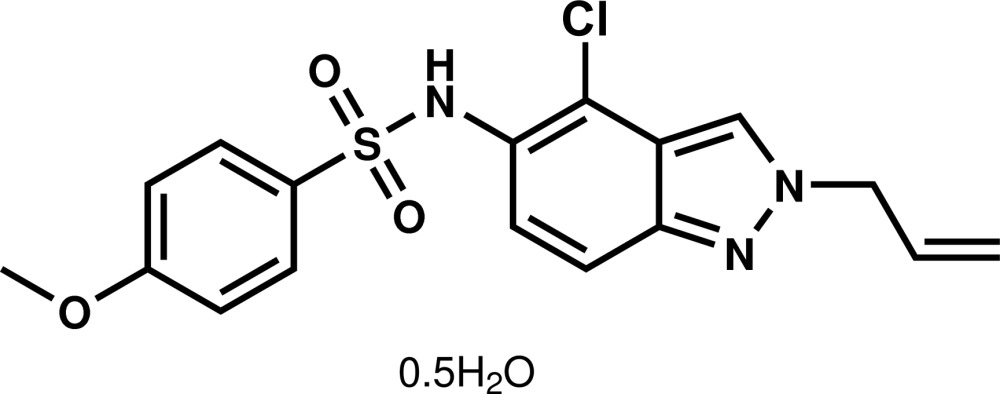


## Experimental

### 

#### Crystal data

C_17_H_16_ClN_3_O_3_S·0.5H_2_O
*M* *_r_* = 386.86
Monoclinic, 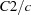

*a* = 23.5515 (9) Å
*b* = 8.9081 (3) Å
*c* = 20.8278 (8) Å
β = 122.628 (2)°
*V* = 3680.1 (2) Å^3^
*Z* = 8
Mo *K*α radiation
μ = 0.35 mm^−1^
*T* = 296 K
0.41 × 0.38 × 0.27 mm

#### Data collection

Bruker X8 APEX diffractometer
20391 measured reflections
4389 independent reflections
2740 reflections with *I* > 2σ(*I*)
*R*_int_ = 0.057

#### Refinement

*R*[*F* ^2^ > 2σ(*F* ^2^)] = 0.047
*wR*(*F* ^2^) = 0.122
*S* = 1.02
4389 reflections
231 parameters
H-atom parameters constrained
Δρ_max_ = 0.34 e Å^−3^
Δρ_min_ = −0.33 e Å^−3^

### 

Data collection: *APEX2* (Bruker, 2009 ▶); cell refinement: *SAINT* (Bruker, 2009 ▶); data reduction: *SAINT*; program(s) used to solve structure: *SHELXS97* (Sheldrick, 2008 ▶); program(s) used to refine structure: *SHELXL97* (Sheldrick, 2008 ▶); molecular graphics: *ORTEP-3 for Windows* (Farrugia, 2012 ▶); software used to prepare material for publication: *PLATON* (Spek, 2009 ▶) and *publCIF* (Westrip, 2010 ▶).

## Supplementary Material

Crystal structure: contains datablock(s) I. DOI: 10.1107/S1600536813020606/tk5244sup1.cif

Structure factors: contains datablock(s) I. DOI: 10.1107/S1600536813020606/tk5244Isup2.hkl

Supplementary material file. DOI: 10.1107/S1600536813020606/tk5244Isup3.cml

Additional supplementary materials:  crystallographic information; 3D view; checkCIF report

## Figures and Tables

**Table 1:** Hydrogen-bond geometry (Å, °)

*D*—H⋯*A*	*D*—H	H⋯*A*	*D*⋯*A*	*D*—H⋯*A*
N1—H1⋯O4^i^	0.83	2.04	2.875 (2)	174
O4—H4⋯N2	0.87	2.00	2.822 (2)	158
C7—H7⋯O3^ii^	0.93	2.37	3.288 (3)	170

Symmetry codes: (i) 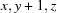; (ii) 
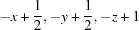
.

**Fig. 1.**
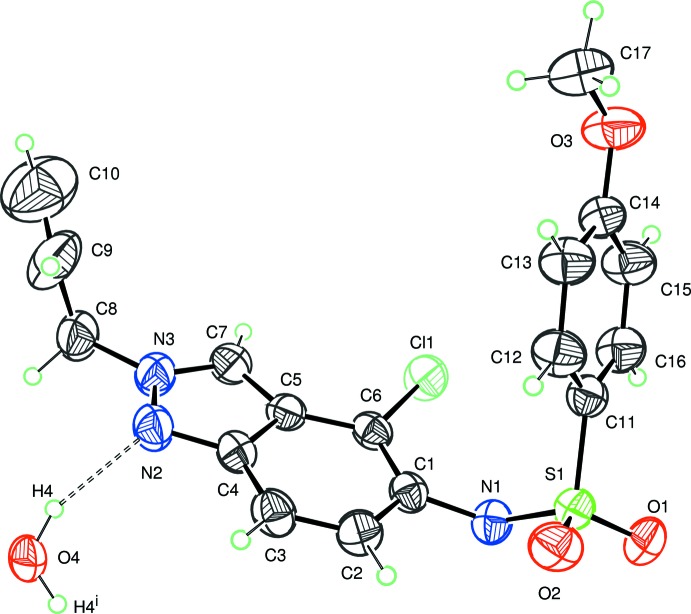
Molecular structure of the title compound with the atom-labelling scheme. Displacement ellipsoids are drawn at the 50% probability level. H atoms are represented as small circles.

## supplementary crystallographic information

### Comment

Research on sulfonamides are justified by the medical interest of these compounds. Indeed, these drugs possess different types of pharmacological activities such as anti-bacterial, hypoglycemic, anti-inflammatory agents and anti-tumour (Lopez, *et al.*, 2010), anti-carbonic anhydrase (Brzozowski *et al.*, 2010), hypoglycemia (Drew, 2000) and anti-cancer activity (Garaj *et al.*, 2005). This work is part of research on the synthesis of some new *N*-(6 (4)-indazolyl derivatives)aylsulfonamide recently reported by our group (Abbassi *et al.*, 2012, Abbassi *et al.*, 2013).

The molecule of the *N*-(2-allyl-5-chloro-2*H*-indazol-5-yl)-4-methoxybenzenesulfonamide is built up from fused five- and six-membered rings which are almost co-planar, with a maximum deviation of -0.057 (3) Å for C1 atom as shown in Fig. 1. Moreover, the fused rings system is nearly perpendicular to the plane through the atoms forming the allyl group (C8-C10) and to benzene ring (C11-C16) as indicated by the dihedral angles between them of 66.4 (5) and 66.18 (12)°, respectively.

The cohesion of the crystal structure is ensured by O4–H4···N2, N1–H1···O4 and C7–H7···O3 hydrogen bonds formed between the water and the organic molecules forming a one-dimensional chain along the *b* axis (Table 2).

### Experimental

A mixture of 2-allyl-5-nitroindazole (1.22 mmol) and anhydrous SnCl_2_ (1.1 g, 6.1 mmol) in absolute ethanol (25 ml) was heated at 333 K for 6 h. After reduction, the starting material disappeared, and the solution was allowed to cool down. The pH was made slightly basic (pH 7–8) by addition of 5% aqueous potassium bicarbonate before extraction with ethyl acetate. The organic phase was washed with brine and dried over magnesium sulfate. The solvent was removed to afford the amine, which was immediately dissolved in pyridine (5 ml) and then reacted with 4-methoxybenzenesulfonyl chloride (1.25 mmol) at room temperature for 24 h. After the reaction mixture was concentrated in *vacuo*, the resulting residue was purified by flash chromatography (eluted with ethyl acetate:hexane 1:9). The title compound was recrystallized from its ethanol solution.

### Refinement

The C-bound H atoms were located in a difference map and treated as riding with C—H = 0.93-0.97 Å for methyl-, methylene-, aromatic-H, respectively. The N—H and O–H atoms were included in their "as located" positions. *U*_iso_(H) = 1.2*U*_eq_ (aromatic, methylene, NH and OH) and *U*_iso_(H) = 1.5 *U*_eq_(methyl).

### Crystal data

**Table:**

C_17_H_16_ClN_3_O_3_S·0.5H_2_O	*F*(000) = 1608
*M**_r_* = 386.86	*D*_x_ = 1.396 Mg m^−^^3^
Monoclinic, *C*2/*c*	Mo *K*α radiation, λ = 0.71073 Å
Hall symbol: -C 2yc	Cell parameters from 4389 reflections
*a* = 23.5515 (9) Å	θ = 2.5–27.9°
*b* = 8.9081 (3) Å	µ = 0.35 mm^−^^1^
*c* = 20.8278 (8) Å	*T* = 296 K
β = 122.628 (2)°	Block, colourless
*V* = 3680.1 (2) Å^3^	0.41 × 0.38 × 0.27 mm
*Z* = 8	

### Data collection

**Table:**

Bruker X8 APEX diffractometer	2740 reflections with *I* > 2σ(*I*)
Radiation source: fine-focus sealed tube	*R*_int_ = 0.057
Graphite monochromator	θ_max_ = 27.9°, θ_min_ = 2.5°
φ and ω scans	*h* = −30→29
20391 measured reflections	*k* = −11→11
4389 independent reflections	*l* = −25→27

### Refinement

**Table:**

Refinement on *F*^2^	Primary atom site location: structure-invariant direct methods
Least-squares matrix: full	Secondary atom site location: difference Fourier map
*R*[*F*^2^ > 2σ(*F*^2^)] = 0.047	Hydrogen site location: inferred from neighbouring sites
*wR*(*F*^2^) = 0.122	H-atom parameters constrained
*S* = 1.02	*w* = 1/[σ^2^(*F*_o_^2^) + (0.0457*P*)^2^ + 2.416*P*] where *P* = (*F*_o_^2^ + 2*F*_c_^2^)/3
4389 reflections	(Δ/σ)_max_ < 0.001
231 parameters	Δρ_max_ = 0.34 e Å^−^^3^
0 restraints	Δρ_min_ = −0.33 e Å^−^^3^

### Special details

**Table:**

Geometry. All s.u.'s (except the s.u. in the dihedral angle between two l.s. planes) are estimated using the full covariance matrix. The cell s.u.'s are taken into account individually in the estimation of s.u.'s in distances, angles and torsion angles; correlations between s.u.'s in cell parameters are only used when they are defined by crystal symmetry. An approximate (isotropic) treatment of cell s.u.'s is used for estimating s.u.'s involving l.s. planes.
Refinement. Refinement of *F*^2^ against all reflections. The weighted *R*-factor *wR* and goodness of fit *S* are based on *F*^2^, conventional *R*-factors *R* are based on *F*, with *F* set to zero for negative *F*^2^. The threshold expression of *F*^2^ > 2σ(*F*^2^) is used only for calculating *R*-factors(gt) *etc*. and is not relevant to the choice of reflections for refinement. *R*-factors based on *F*^2^ are statistically about twice as large as those based on *F*, and *R*- factors based on all data will be even larger.

### Fractional atomic coordinates and isotropic or equivalent isotropic displacement parameters (Å^2^)

**Table:**

	*x*	*y*	*z*	*U*_iso_*/*U*_eq_	
C1	0.07686 (11)	0.1101 (2)	0.24133 (13)	0.0363 (5)	
C2	0.06759 (12)	−0.0223 (3)	0.19808 (14)	0.0434 (6)	
H2	0.0569	−0.0121	0.1483	0.052*	
C3	0.07383 (13)	−0.1630 (3)	0.22687 (14)	0.0457 (6)	
H3	0.0688	−0.2476	0.1981	0.055*	
C4	0.08816 (12)	−0.1761 (3)	0.30157 (13)	0.0402 (5)	
C5	0.09891 (11)	−0.0468 (2)	0.34607 (13)	0.0369 (5)	
C6	0.09541 (11)	0.0968 (2)	0.31577 (13)	0.0361 (5)	
C7	0.10847 (12)	−0.1007 (3)	0.41405 (14)	0.0458 (6)	
H7	0.1169	−0.0441	0.4558	0.055*	
C8	0.11049 (16)	−0.3571 (3)	0.46422 (16)	0.0612 (8)	
H8A	0.0770	−0.4358	0.4394	0.073*	
H8B	0.1028	−0.3065	0.5001	0.073*	
C9	0.1797 (2)	−0.4252 (4)	0.5063 (2)	0.0854 (11)	
H9	0.1947	−0.4679	0.4773	0.103*	
C10	0.2188 (2)	−0.4290 (6)	0.5770 (3)	0.1285 (17)	
H10A	0.2056	−0.3874	0.6080	0.154*	
H10B	0.2610	−0.4734	0.5987	0.154*	
C11	0.19083 (12)	0.3430 (3)	0.25760 (13)	0.0404 (5)	
C12	0.23937 (14)	0.2387 (3)	0.27121 (15)	0.0517 (7)	
H12	0.2288	0.1607	0.2369	0.062*	
C13	0.30364 (14)	0.2495 (3)	0.33557 (15)	0.0521 (7)	
H13	0.3364	0.1796	0.3443	0.062*	
C14	0.31891 (13)	0.3638 (3)	0.38665 (14)	0.0448 (6)	
C15	0.26970 (14)	0.4682 (3)	0.37284 (15)	0.0555 (7)	
H15	0.2802	0.5461	0.4072	0.067*	
C16	0.20606 (13)	0.4575 (3)	0.30924 (15)	0.0514 (7)	
H16	0.1732	0.5269	0.3008	0.062*	
C17	0.43186 (15)	0.2760 (4)	0.47218 (17)	0.0706 (9)	
H17A	0.4722	0.3057	0.5189	0.106*	
H17B	0.4409	0.2688	0.4325	0.106*	
H17C	0.4171	0.1802	0.4790	0.106*	
N1	0.06279 (10)	0.2526 (2)	0.20483 (11)	0.0415 (5)	
H1	0.0473	0.3177	0.2200	0.050*	
N2	0.09126 (11)	−0.3021 (2)	0.33985 (12)	0.0491 (5)	
N3	0.10311 (11)	−0.2499 (2)	0.40710 (12)	0.0481 (5)	
O1	0.08137 (9)	0.47240 (19)	0.14958 (10)	0.0602 (5)	
O2	0.11154 (9)	0.2212 (2)	0.12606 (10)	0.0550 (5)	
O3	0.38047 (10)	0.3849 (2)	0.45159 (10)	0.0651 (6)	
O4	0.0000	−0.5316 (2)	0.2500	0.0455 (6)	
H4	0.0324	−0.4800	0.2868	0.055*	
S1	0.10886 (3)	0.32522 (7)	0.17658 (3)	0.04380 (18)	
Cl1	0.11387 (4)	0.25023 (7)	0.37487 (4)	0.0539 (2)	

### Atomic displacement parameters (Å^2^)

**Table:**

	*U*^11^	*U*^22^	*U*^33^	*U*^12^	*U*^13^	*U*^23^
C1	0.0325 (12)	0.0362 (11)	0.0432 (14)	−0.0015 (9)	0.0224 (10)	−0.0012 (10)
C2	0.0502 (15)	0.0461 (13)	0.0395 (14)	−0.0016 (11)	0.0279 (12)	−0.0045 (11)
C3	0.0548 (15)	0.0385 (13)	0.0473 (15)	−0.0058 (11)	0.0299 (13)	−0.0112 (11)
C4	0.0418 (13)	0.0355 (12)	0.0426 (13)	−0.0036 (10)	0.0223 (11)	−0.0035 (10)
C5	0.0334 (12)	0.0374 (12)	0.0354 (12)	−0.0021 (10)	0.0156 (10)	−0.0031 (10)
C6	0.0328 (12)	0.0341 (11)	0.0399 (13)	−0.0014 (9)	0.0186 (10)	−0.0063 (10)
C7	0.0488 (15)	0.0420 (13)	0.0425 (14)	−0.0026 (11)	0.0220 (12)	−0.0046 (11)
C8	0.078 (2)	0.0522 (15)	0.0503 (16)	−0.0095 (15)	0.0324 (15)	0.0060 (13)
C9	0.113 (3)	0.069 (2)	0.060 (2)	0.022 (2)	0.038 (2)	0.0236 (18)
C10	0.090 (3)	0.168 (5)	0.130 (4)	0.029 (3)	0.061 (3)	0.046 (4)
C11	0.0413 (13)	0.0437 (13)	0.0389 (13)	−0.0031 (11)	0.0233 (11)	0.0003 (11)
C12	0.0509 (16)	0.0530 (15)	0.0490 (16)	−0.0001 (12)	0.0255 (13)	−0.0111 (12)
C13	0.0469 (15)	0.0546 (15)	0.0521 (16)	0.0088 (12)	0.0250 (13)	−0.0044 (13)
C14	0.0446 (14)	0.0488 (14)	0.0360 (13)	−0.0008 (11)	0.0185 (11)	0.0006 (11)
C15	0.0555 (17)	0.0510 (15)	0.0479 (16)	0.0041 (13)	0.0200 (13)	−0.0123 (12)
C16	0.0516 (16)	0.0462 (14)	0.0530 (16)	0.0083 (12)	0.0259 (13)	−0.0020 (12)
C17	0.0550 (18)	0.085 (2)	0.0531 (18)	0.0131 (16)	0.0170 (15)	0.0047 (16)
N1	0.0426 (11)	0.0384 (10)	0.0480 (12)	0.0044 (9)	0.0272 (10)	0.0030 (9)
N2	0.0621 (14)	0.0374 (10)	0.0467 (13)	−0.0070 (10)	0.0285 (11)	−0.0033 (10)
N3	0.0580 (14)	0.0422 (11)	0.0400 (12)	−0.0039 (10)	0.0238 (11)	0.0007 (10)
O1	0.0570 (11)	0.0533 (11)	0.0583 (12)	0.0038 (9)	0.0232 (10)	0.0218 (9)
O2	0.0580 (12)	0.0709 (12)	0.0423 (10)	−0.0083 (9)	0.0311 (9)	−0.0085 (9)
O3	0.0547 (12)	0.0710 (12)	0.0449 (11)	0.0067 (10)	0.0106 (9)	−0.0085 (9)
O4	0.0490 (14)	0.0278 (11)	0.0537 (15)	0.000	0.0238 (12)	0.000
S1	0.0435 (3)	0.0467 (3)	0.0391 (3)	−0.0030 (3)	0.0210 (3)	0.0045 (3)
Cl1	0.0702 (5)	0.0371 (3)	0.0480 (4)	0.0009 (3)	0.0275 (3)	−0.0084 (3)

### Geometric parameters (Å, º)

**Table:**

C1—C6	1.371 (3)	C11—C12	1.379 (3)
C1—N1	1.423 (3)	C11—C16	1.382 (3)
C1—C2	1.428 (3)	C11—S1	1.757 (2)
C2—C3	1.363 (3)	C12—C13	1.382 (4)
C2—H2	0.9300	C12—H12	0.9300
C3—C4	1.408 (3)	C13—C14	1.373 (3)
C3—H3	0.9300	C13—H13	0.9300
C4—N2	1.356 (3)	C14—O3	1.361 (3)
C4—C5	1.413 (3)	C14—C15	1.390 (4)
C5—C7	1.393 (3)	C15—C16	1.367 (3)
C5—C6	1.409 (3)	C15—H15	0.9300
C6—Cl1	1.731 (2)	C16—H16	0.9300
C7—N3	1.336 (3)	C17—O3	1.425 (3)
C7—H7	0.9300	C17—H17A	0.9600
C8—N3	1.461 (3)	C17—H17B	0.9600
C8—C9	1.501 (5)	C17—H17C	0.9600
C8—H8A	0.9700	N1—S1	1.622 (2)
C8—H8B	0.9700	N1—H1	0.8338
C9—C10	1.247 (5)	N2—N3	1.355 (3)
C9—H9	0.9300	O1—S1	1.4359 (18)
C10—H10A	0.9300	O2—S1	1.4286 (18)
C10—H10B	0.9300	O4—H4	0.8662
			
C6—C1—N1	121.5 (2)	C11—C12—C13	120.4 (2)
C6—C1—C2	119.3 (2)	C11—C12—H12	119.8
N1—C1—C2	119.2 (2)	C13—C12—H12	119.8
C3—C2—C1	122.6 (2)	C14—C13—C12	119.7 (2)
C3—C2—H2	118.7	C14—C13—H13	120.2
C1—C2—H2	118.7	C12—C13—H13	120.2
C2—C3—C4	117.9 (2)	O3—C14—C13	124.5 (2)
C2—C3—H3	121.1	O3—C14—C15	115.9 (2)
C4—C3—H3	121.1	C13—C14—C15	119.7 (2)
N2—C4—C3	128.5 (2)	C16—C15—C14	120.7 (2)
N2—C4—C5	110.8 (2)	C16—C15—H15	119.7
C3—C4—C5	120.6 (2)	C14—C15—H15	119.7
C7—C5—C6	134.9 (2)	C15—C16—C11	119.6 (2)
C7—C5—C4	105.0 (2)	C15—C16—H16	120.2
C6—C5—C4	120.0 (2)	C11—C16—H16	120.2
C1—C6—C5	119.4 (2)	O3—C17—H17A	109.5
C1—C6—Cl1	122.73 (17)	O3—C17—H17B	109.5
C5—C6—Cl1	117.83 (18)	H17A—C17—H17B	109.5
N3—C7—C5	106.2 (2)	O3—C17—H17C	109.5
N3—C7—H7	126.9	H17A—C17—H17C	109.5
C5—C7—H7	126.9	H17B—C17—H17C	109.5
N3—C8—C9	110.7 (3)	C1—N1—S1	122.73 (16)
N3—C8—H8A	109.5	C1—N1—H1	116.2
C9—C8—H8A	109.5	S1—N1—H1	112.1
N3—C8—H8B	109.5	N3—N2—C4	103.84 (18)
C9—C8—H8B	109.5	C7—N3—N2	114.1 (2)
H8A—C8—H8B	108.1	C7—N3—C8	126.8 (2)
C10—C9—C8	125.3 (4)	N2—N3—C8	119.0 (2)
C10—C9—H9	117.4	C14—O3—C17	118.8 (2)
C8—C9—H9	117.4	O2—S1—O1	119.55 (12)
C9—C10—H10A	120.0	O2—S1—N1	108.20 (11)
C9—C10—H10B	120.0	O1—S1—N1	104.94 (11)
H10A—C10—H10B	120.0	O2—S1—C11	107.69 (12)
C12—C11—C16	119.9 (2)	O1—S1—C11	108.89 (11)
C12—C11—S1	119.78 (19)	N1—S1—C11	106.92 (11)
C16—C11—S1	120.25 (19)		
			
C6—C1—C2—C3	2.7 (4)	C13—C14—C15—C16	0.6 (4)
N1—C1—C2—C3	−173.7 (2)	C14—C15—C16—C11	−1.0 (4)
C1—C2—C3—C4	1.8 (4)	C12—C11—C16—C15	1.3 (4)
C2—C3—C4—N2	174.2 (2)	S1—C11—C16—C15	178.7 (2)
C2—C3—C4—C5	−3.0 (4)	C6—C1—N1—S1	114.3 (2)
N2—C4—C5—C7	−0.3 (3)	C2—C1—N1—S1	−69.4 (3)
C3—C4—C5—C7	177.4 (2)	C3—C4—N2—N3	−176.8 (2)
N2—C4—C5—C6	−177.8 (2)	C5—C4—N2—N3	0.6 (3)
C3—C4—C5—C6	−0.1 (3)	C5—C7—N3—N2	0.6 (3)
N1—C1—C6—C5	170.4 (2)	C5—C7—N3—C8	178.8 (2)
C2—C1—C6—C5	−5.8 (3)	C4—N2—N3—C7	−0.8 (3)
N1—C1—C6—Cl1	−8.6 (3)	C4—N2—N3—C8	−179.2 (2)
C2—C1—C6—Cl1	175.13 (17)	C9—C8—N3—C7	−99.0 (3)
C7—C5—C6—C1	−171.9 (3)	C9—C8—N3—N2	79.2 (3)
C4—C5—C6—C1	4.6 (3)	C13—C14—O3—C17	−5.1 (4)
C7—C5—C6—Cl1	7.2 (4)	C15—C14—O3—C17	175.5 (3)
C4—C5—C6—Cl1	−176.30 (18)	C1—N1—S1—O2	56.3 (2)
C6—C5—C7—N3	176.7 (3)	C1—N1—S1—O1	−175.03 (18)
C4—C5—C7—N3	−0.2 (3)	C1—N1—S1—C11	−59.5 (2)
N3—C8—C9—C10	127.3 (4)	C12—C11—S1—O2	−15.3 (2)
C16—C11—C12—C13	−1.2 (4)	C16—C11—S1—O2	167.2 (2)
S1—C11—C12—C13	−178.6 (2)	C12—C11—S1—O1	−146.4 (2)
C11—C12—C13—C14	0.7 (4)	C16—C11—S1—O1	36.2 (2)
C12—C13—C14—O3	−179.9 (3)	C12—C11—S1—N1	100.7 (2)
C12—C13—C14—C15	−0.4 (4)	C16—C11—S1—N1	−76.7 (2)
O3—C14—C15—C16	−180.0 (3)		

### Hydrogen-bond geometry (Å, º)

**Table:**

*D*—H···*A*	*D*—H	H···*A*	*D*···*A*	*D*—H···*A*
N1—H1···O4^i^	0.83	2.04	2.875 (2)	174
O4—H4···N2	0.87	2.00	2.822 (2)	158
C7—H7···O3^ii^	0.93	2.37	3.288 (3)	170

Symmetry codes: (i) *x*, *y*+1, *z*; (ii) −*x*+1/2, −*y*+1/2, −*z*+1.

## References

[bb1] Abbassi, N., Chicha, H., Rakib, E. M., Hannioui, A., Alaoui, M., Hajjaji, A., Geffken, D., Aiello, C., Gangemi, R., Rosano, C. & Viale, M. (2012). *Eur. J. Med. Chem.* **57**, 240–249. 10.1016/j.ejmech.2012.09.013 23072738

[bb2] Abbassi, N., Rakib, E. M., Hannioui, A., Saadi, M. & El Ammari, L. (2013). *Acta Cryst.* E**69**, o190–o191. 10.1107/S1600536812051975 PMC3569252 23424475

[bb3] Bruker (2009). *APEX2*, *SAINT* and *SADABS* Bruker AXS Inc., Madison, Wisconsin, USA.

[bb4] Brzozowski, Z., Slawinski, J., Saczewski, F., Innocenti, A. & Supuran, C. T. (2010). *Eur. J. Med. Chem.* **45**, 2396–2404. 10.1016/j.ejmech.2010.02.020 20202722

[bb5] Drew, J. (2000). *Science*, **287**, 1960–964.

[bb6] Farrugia, L. J. (2012). *J. Appl. Cryst.* **45**, 849–854.

[bb7] Garaj, V., Puccetti, L., Fasolis, G., Winum, J. Y., Montero, J. L., Scozzafava, A., Vullo, D., Innocenti, A. & Supuran, C. T. (2005). *Bioorg. Med. Chem. Lett.* **15**, 3102–3108. 10.1016/j.bmcl.2005.04.056 15905091

[bb8] Lopez, M., Bornaghi, L. F., Innocenti, A., Vullo, D., Charman, S. A., Supuran, C. T. & Poulsen, S.-A. (2010). *J. Med. Chem.* **53**, 2913–2926. 10.1021/jm901888x 20201556

[bb9] Sheldrick, G. M. (2008). *Acta Cryst.* A**64**, 112–122. 10.1107/S0108767307043930 18156677

[bb10] Spek, A. L. (2009). *Acta Cryst.* D**65**, 148–155. 10.1107/S090744490804362X PMC2631630 19171970

[bb11] Westrip, S. P. (2010). *J. Appl. Cryst.* **43**, 920–925.

